# Beyond trauma: a case-based imaging review of spontaneous splenic rupture

**DOI:** 10.3389/fradi.2025.1715806

**Published:** 2026-01-12

**Authors:** Federica Romano, Marina Alessandrella, Raffaella Lucci, Giorgio Bocchini, Mariano Scaglione, Stefania Tamburrini, Emanuele Muto, Giuseppina Dell’Aversano Orabona, Rosita Comune, Francesco Tiralongo, Graziella Di Grezia, Salvatore Masala, Giacomo Sica

**Affiliations:** 1 Radiology Unit, Monaldi Hospital, Azienda Ospedaliera dei Colli, Naples, Italy; 2 Department of Precision Medicine, University of Campania “L. Vanvitelli”, Naples, Italy; 3 Department of Pathology, Monaldi Hospital, Azienda Ospedaliera dei Colli, Naples, Italy; 4 Radiology Department of Surgery, Medicine and Pharmacy, University of Sassari, Sassari, Italy; 5 Department of Radiology, Ospedale del Mare-ASL NA1 Centro-Napoli, Naples, Italy; 6 Emicenter Srl, Naples, Italy; 7 Department of General and Emergency Radiology, “Antonio Cardarelli” Hospital, Naples, Italy; 8 Radiology Unit 1, Department of Medical Surgical Sciences and Advanced Technologies “GF Ingrassia”, University Hospital Policlinico “G. Rodolico-San Marco”, Catania, Italy; 9 Department of Life Sciences, Health, and Healthcare Professions Link Campus University, Rome, Italy

**Keywords:** AI, computed tomography, emergency radiology, non-operative management, spleen

## Abstract

Spontaneous splenic rupture (SSR) is a rare but potentially life-threatening condition, most commonly associated with underlying infectious, haematological, vascular, or neoplastic processes. Clinical presentation is often non-specific, which may lead to delayed diagnosis. Imaging, particularly contrast-enhanced computed tomography (CECT), plays a pivotal role in confirming splenic injury, identifying predisposing lesions, and guiding management. We present the case of a woman aged in her seventies with chronic atrial fibrillation on antiplatelet therapy who developed spontaneous splenic rupture secondary to an occult splenic hamartoma. Ultrasound demonstrated heterogeneous perisplenic fluid and altered splenic echotexture. CT showed a 3.5 cm laceration, moderate haemoperitoneum, and a solid lesion with delayed enhancement and no active bleeding. Follow-up CT revealed progressive organisation of haemoperitoneum and stable lesion morphology. The patient was initially managed non-operatively due to haemodynamic stability, but elective splenectomy was performed given the presence of a structural lesion and the need for chronic anticoagulation. The purpose of this article is to illustrate the diagnostic and management principles of SSR through a representative clinical case and to provide an updated review of imaging strategies, including emerging applications of radiomics and artificial intelligence (AI).

## Introduction

Abnormalities of the spleen are frequently detected incidentally during imaging studies, especially in patients without clear clinical symptoms. Spontaneous non-traumatic splenic rupture is a rare but significant clinical occurrence, often linked to a spectrum of immunological and non-immunological disorders, which complicates diagnosis. The spleen, responsible for both immunological and haematopoietic functions, is vulnerable to various pathological conditions, including infections, immune-mediated disorders, and neoplastic diseases. These conditions can compromise the structural integrity of the spleen, rendering it more susceptible to rupture (1).

Spontaneous splenic rupture (SSR) typically occurs in the context of underlying pathology, such as infections, haematological disorders, or vascular abnormalities that alter the spleen's architecture (Table 1). Radiological findings, especially those from computed tomography (CT), play a central role in the diagnosis of splenic rupture, but may not always be definitive without careful clinical correlation. Although widely used as a first-line imaging modality, ultrasound is limited by artefacts caused by intestinal air, operator dependency, and challenges in visualising the entire spleen, particularly when it is subcostal or in the presence of diaphragmatic elevation. In contrast, CT imaging offers superior sensitivity for detecting splenic lesions, assessing their extent, and evaluating associated complications, such as hemoperitoneum. Magnetic resonance imaging (MRI) may also be utilised in selected cases for further assessment (2).

**Table 1:** Main causes of spontaneous splenic rupture.

Cause of Spontaneous Splenic Rupture	Description	Frequency	References
Splenic Hamartoma	A rare benign lesion of splenic tissue that can cause rupture due to its fragile vascular architecture.	<1%	Barnes et al. (10)
Malaria	Infection that causes splenomegaly and structural damage to the spleen, making it prone to rupture.	10%–15%	Sterlacci et al. (1)
Hematological Disorders (e.g., leukemia, lymphoma)	Blood disorders that alter the spleen's structure and predispose it to spontaneous rupture.	10%–20%	Mainenti et al. (2)
			Aubrey Bassler (9)
Vasculitis	Autoimmune or inflammatory conditions affecting blood vessels in the spleen, leading to rupture.	5%–10%	Sterlacci et al. (1);
			Thippavong et al. (16)
Splenomegaly	Enlarged spleen making it more susceptible to rupture.	20%–25%	Sterlacci et al. (1)
			Aubrey Bassler (9)
Viral Infections (e.g., Epstein–Barr, Citomegalovirus)	Infections that cause splenic inflammation, making it more vulnerable to rupture.	5%–10%	Thipphavong et al. (16)
Splenic Vascular Malformations	Abnormal blood vessel structures within the spleen that can lead to rupture.	5%–10%	Sterlacci et al. (1)
			Lin JL (18)
Anticoagulants and Antiplatelet Drugs	Medications that alter coagulation, increasing the risk of splenic rupture.	5%–10%	Aubrey Bassler (9)
Neoplastic Splenic Diseases (e.g., metastases)	Tumors or metastases affecting the spleen, making it more prone to rupture.	1%–5%	Sterlacci et al. (1)
			Ballardini P (6)

Reported frequencies are approximate and derived from heterogeneous case series of varying sample size; values should therefore be interpreted as indicative rather than definitive.

Emerging technologies such as artificial intelligence (AI) and radiomics have shown substantial potential in enhancing diagnostic precision. Radiomics, which involves the extraction of large amounts of quantitative features from medical images, can offer new insights into lesion characteristics and heterogeneity, providing valuable information for early diagnosis and risk stratification in splenic pathology. The integration of AI algorithms with radiomic data promises to refine imaging interpretation, offering radiologists powerful tools for identifying splenic lesions that might otherwise remain undetected (3, 4).

## Case presentation

A 75-year-old woman with a history of chronic atrial fibrillation, managed with long-term antiplatelet therapy, presented to the cardiology outpatient clinic at our institution with persistent left flank pain for several days. She denied any history of recent trauma or systemic symptoms. Laboratory tests revealed mild anemia (haemoglobin 9.7 g/dL), a preserved platelet count and a prolonged aPTT as INR, attributable to chronic antiplatelet therapy. Clinical examination revealed no visible ecchymosis over the left flank. Deep palpation of the left hypochondrial region elicited significant tenderness and a palpable mass was present, prompting urgent imaging evaluation.

### Initial imaging findings

**Figure 1:**
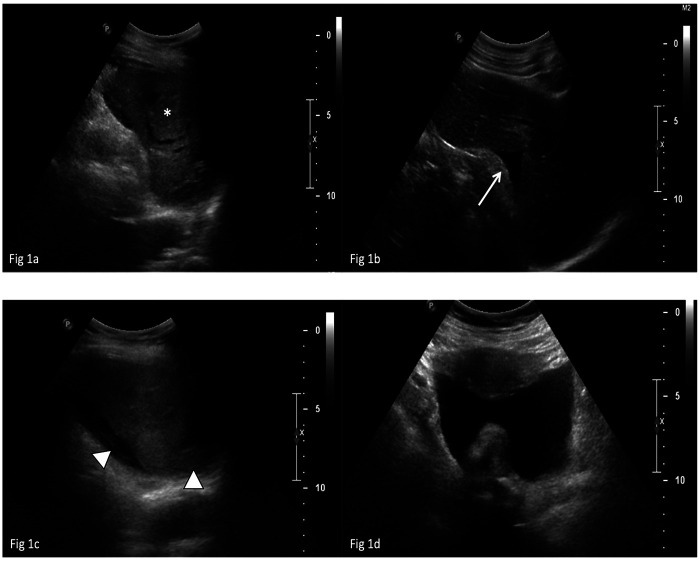
Abdominal ultrasound exam. In **(a)**, a collection with heterogeneous, predominantly anechoic echotexture and hyperechoic strands is observed in the perisplenic region. A thin hypoechoic band surrounds a relatively hyperechoic central splenic area, raising suspicion of a focal lesion (*). In **(b)** and **(c)**, the heterogeneous fluid collection (arrow) surrounds the spleen and contains hyperechoic strands (arrowheads in **c**). In **(d)**, free heterogeneous fluid extends into the pelvic cavity.

**Figure 2:**
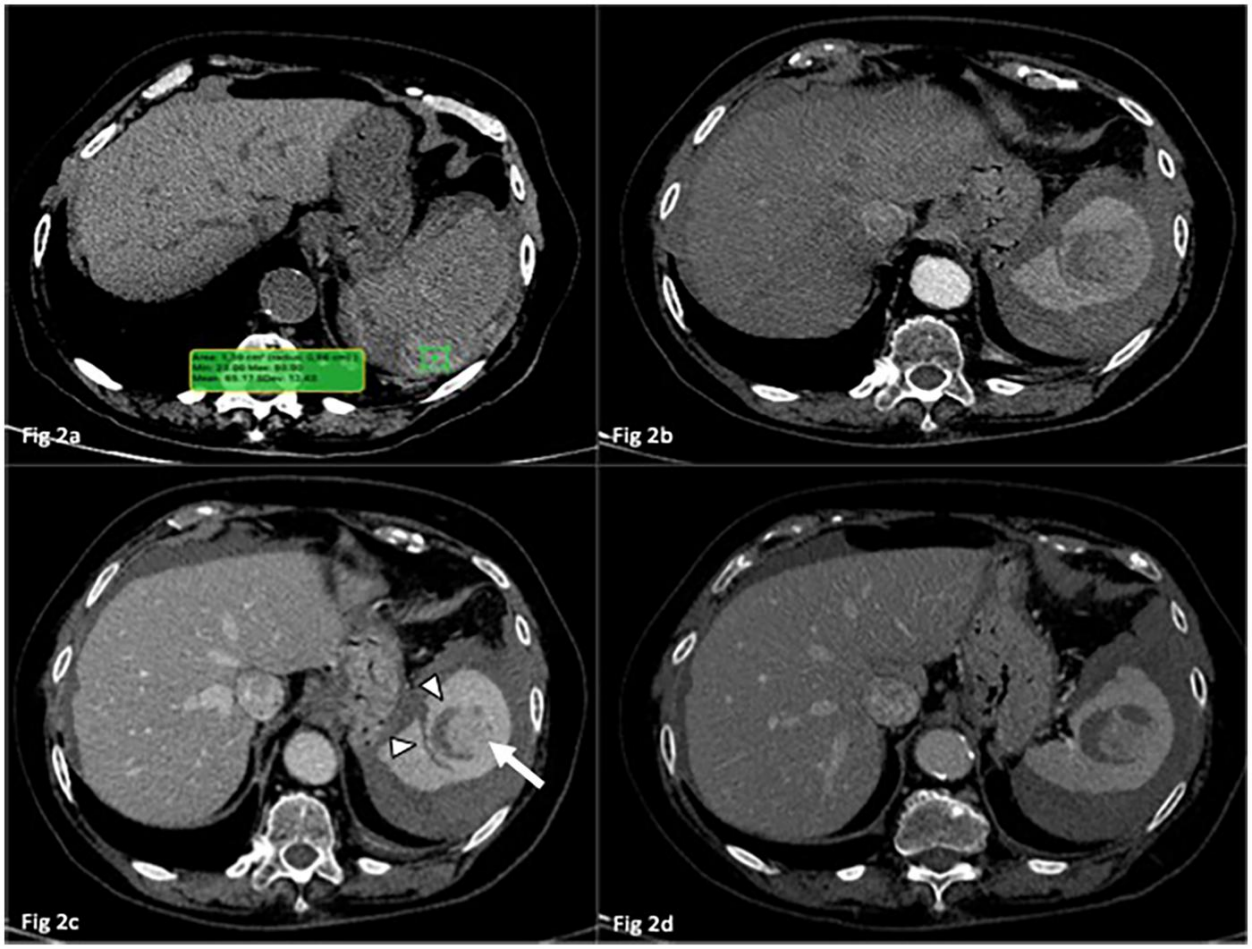
Axial CT scans. In **(a)** baseline CT scan confirms the presence of hemoperitoneum with sentinel clots in the perisplenic region (Region of interest ROI indicates 65HU, Hounsfield Unit). In **(b–d)** a well-defined hypodense laceration (arrowheads in **c**) is observed in continuity with a solid focal alteration (*arrow*), surrounded by a hypodense halo. These findings suggest that the rupture originated from the focal lesion, which may represent the structural substrate for SSR. In all enhanced phases, no evidence of active bleeding is noted.

An abdominal ultrasound (Figures 1a–d) revealed a significant perisplenic fluid collection (4 cm in maximum), with heterogeneous internal echoes highly suggestive of hemoperitoneum. The spleen exhibited an altered echotexture, particularly in the central region, raising suspicion of an underlying structural abnormality. Given the high suspicion of spontaneous splenic rupture, an urgent multiphase contrast-enhanced computed tomography (CECT) scan with angiographic technique was performed. The CT confirmed the presence of moderate hemoperitoneum secondary to a spontaneous splenic laceration. The laceration measured approximately 3,5 cm in depth and 6 mm in width, and was contiguous with a roughly oval lesion within the splenic parenchyma, suggesting a potential predisposing structural weakness leading to the rupture. The lesion demonstrated heterogeneous attenuation, characterized by hypovascular streaks isodense to splenic parenchyma interspersed with slightly hyperdense areas, producing a concentric ring like pattern. Notably, no signs of active arterial extravasation were detected (Figures 2a–d).

### Multidisciplinary management strategy

**Figure 3:**
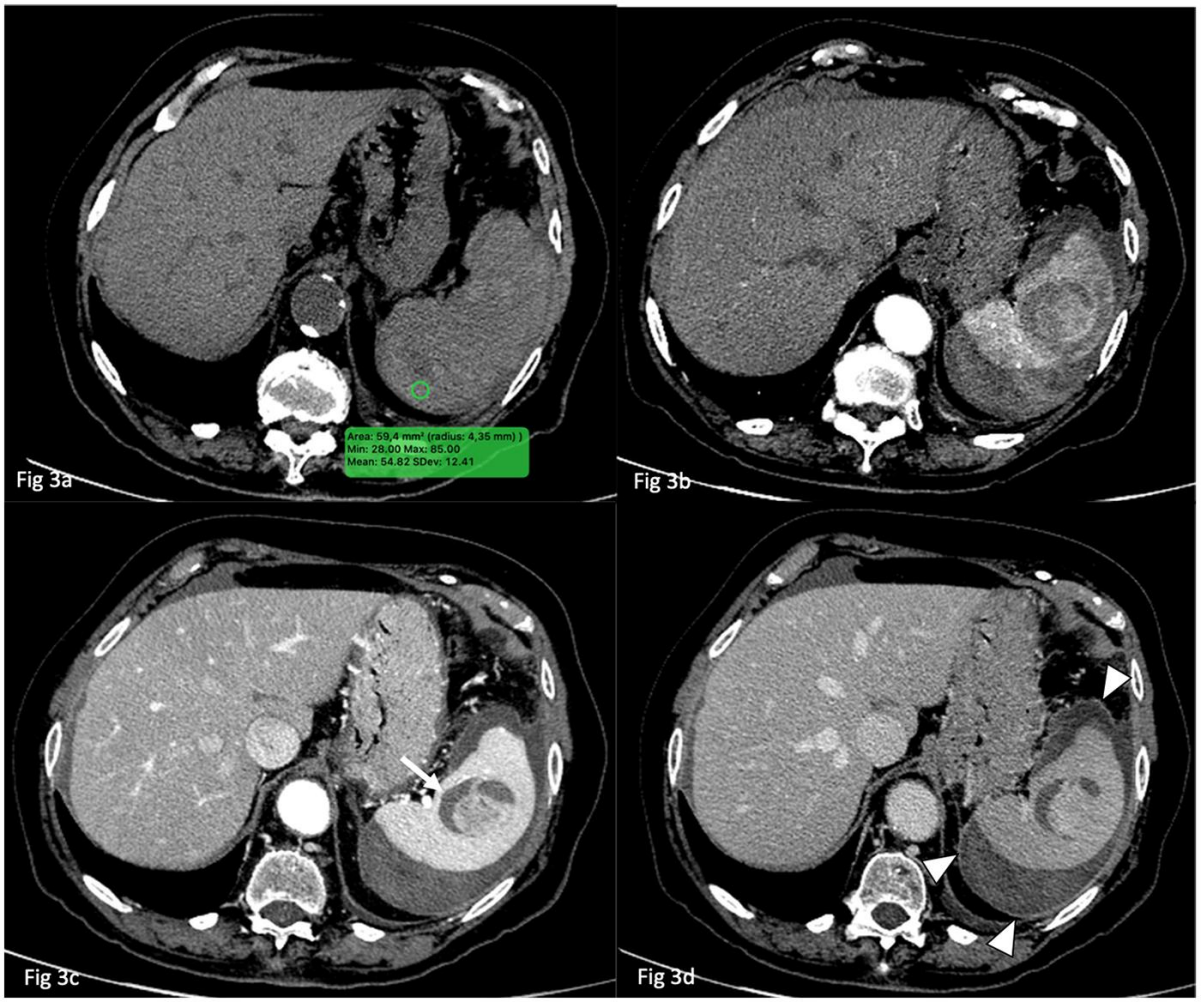
24 h follow-up CT scan. In **(a)** the unenhanced exam shows persistent perisplenic hyperdensity due to hemoperitoneum (*circle ROI*). In **(b,c)** the hemoperitoneum exhibits increased density heterogeneity due to blood degradation products in different phases of hemoglobin breakdown. The hypervascular central splenic lesion and peripheral hypodense halo (*arrow*) remain stable. There are still no signs of active bleeding but in in the late-phase CT axial scan **(d)**, compartmentalization of hemoperitoneum is noted with the formation of a delayed contrast-enhancing wall (arrowheads).

**Figure 4:**
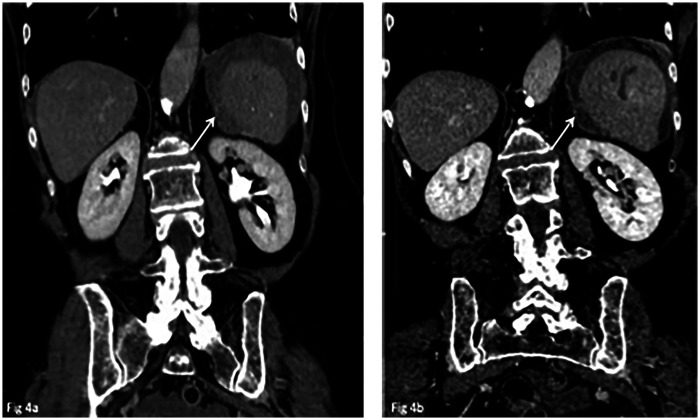
Coronal MPR reconstructions of the late phases illustrate the compartmentalisation of perisplenic haemoperitoneum at the 24 h follow-up CT exam (*arrow in* **b**), which was not clearly visible on the initial CT scan (*arrow in* **a**).

Despite the presence of hemoperitoneum, the patient remained hemodynamically stable. In light of her stability and the absence of ongoing active bleeding, a multidisciplinary team including a radiologist, a surgeon, and an internal medicine specialist opted for initial non-operative management (NOM) based on non-critical imaging findings. The patient was admitted for close monitoring, fluid resuscitation, and correction of coagulation parameters, with serial imaging planned to assess for disease progression. A follow-up CT scan performed 24 h later demonstrated redistribution of the hemoperitoneum and signs of its organization, but no active bleeding was detected (Figures 3, 4). However, elective surgical intervention was recommended due to the compartimentalized haemoperitoneum and the patient's anticipated need for long-term anticoagulant therapy. An elective laparoscopic splenectomy, via laparoscopy, was successfully performed without complications. Intraoperatively, haemoperitoneum was confirmed, with a substantial splenic laceration in close proximity to a solid neoplastic-appearing formation.

### Histopathological and clinical outcomes

**Figure 5:**
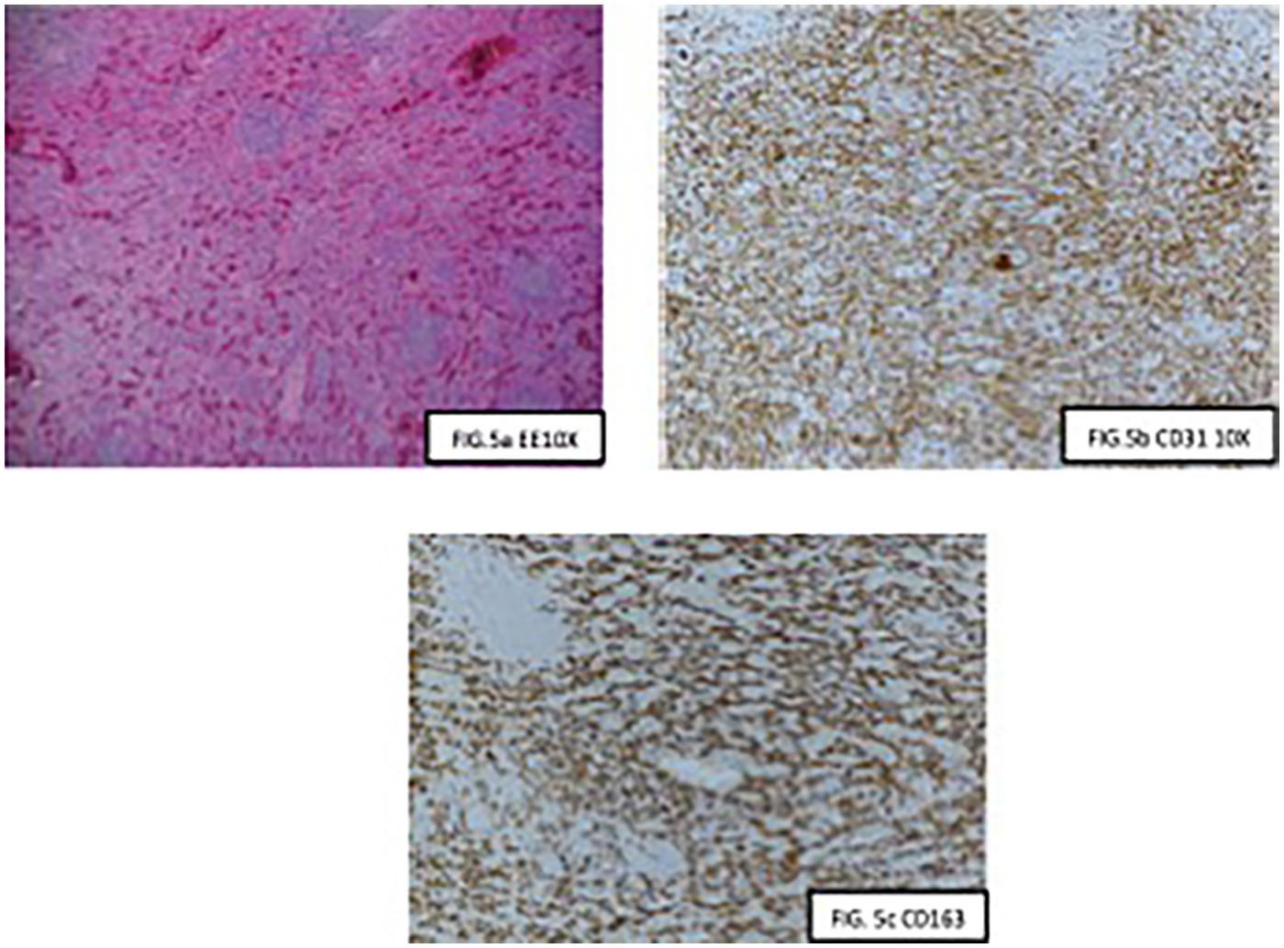
Splenic hamartoma is composed exclusively of red pulp elements **(a)**; the red pulp elements include sinusoids lined by littoral, capillaries lined by usual type endothelium (CD31+) (**b**, 10X) and small veins (CD163+; **c**) the red pulp also contains histiocytes, antigen presenting cells, fibroblasts and pericytes.

Histopathological examination of the resected spleen confirmed the diagnosis of a splenic hamartoma (Figure 5), an uncommon benign vascular lesion that is typically asymptomatic but may predispose the spleen to rupture due to its friable vascular architecture. The patient had an uneventful postoperative course and was discharged in stable condition, after 5 days with no signs of bleeding or infection.

## Discussion

Spontaneous splenic rupture (SSR), while rare, should be considered in the differential diagnosis of patients presenting with unexplained abdominal pain and underlying comorbidities. This condition is often the first clinical manifestation of an underlying pathology, and it is essential for emergency physicians to be aware of its possibility even in the absence of trauma.

The clinical presentation of splenic rupture can be subtle, with symptoms such as abdominal pain, fever, and signs of internal bleeding. Many splenic lesions remain asymptomatic and are detected incidentally during imaging studies performed for unrelated indications. SSR is most commonly seen in patients with underlying conditions such as infections, haematological disorders, or vascular abnormalities, which compromise the structural integrity of the spleen. Risk factors include splenomegaly, vascular malformations, and conditions that predispose to haemodynamic instability.

Sterlacci et al. reported that 90% of splenic ruptures were trauma-related, while 10% were linked to internal diseases (1). Given the potentially high mortality associated with delayed diagnosis, early recognition and prompt management are crucial. Non-traumatic splenic rupture is often associated with conditions such as malaria, lymphoproliferative disorders, and vasculitis.

SSR manifests with diverse clinical features, necessitating vigilant diagnostic scrutiny and expedited imaging confirmation. For instance, Tunçyürek et al. (5) documented a 75-year-old woman presenting with acute abdominal discomfort due to SSR, diagnosed via CT despite the absence of splenic enlargement, highlighting the pivotal role of prompt imaging in such cases. In another report, Ballardini et al. (6) described a 60-year-old man with stage IIIa squamous cell lung carcinoma who experienced SSR shortly after initiating chemotherapy, with CT imaging disclosing significant haemoperitoneum. Similarly, Roll et al. (7) reported a 58-year-old male with infective endocarditis who developed left upper quadrant pain, with CT confirming splenic rupture, thus positioning SSR as a key consideration in patients with chronic inflammatory diseases. Looseley et al. (8) further illustrated this variability, reporting a 22-year-old male with infectious mononucleosis who collapsed secondary to SSR, evidenced by CT detection of intraperitoneal free fluid.

## Aetiological factors and risk profiles

The origins of SSR are multifactorial and complex. Aubrey-Bassler and Sowers (9) conducted a systematic analysis of 613 cases, revealing that 327 were associated with underlying conditions such as infections, haematological malignancies, or neoplasms, while 112 followed medical procedures, notably colonoscopy. Only 35 cases were deemed truly spontaneous, occurring in histologically unremarkable spleens. Infectious causes, exemplified by infectious mononucleosis (8), and haematological disorders, such as amyloidosis linked to prolonged injection drug use (7), emerged as significant contributors. Iatrogenic factors, including chemotherapy (6) and anticoagulant therapy (10), may heighten vascular susceptibility. Moreover, splenic hamartomas, as noted by Seyama et al. (11) and Barnes et al. (10), predispose to rupture due to inherent structural weaknesses, an effect potentially exacerbated by conditions such as portal hypertension or coagulopathy. Aubrey-Bassler and Sowers (9) also identified additional predisposing factors, including pregnancy, anticoagulant or thrombolytic use, and minor physical trauma.

## Splenic hamartoma: entity, frequency, and imaging characteristics

Splenic hamartomas are rare, benign lesions composed of an abnormal proliferation of spleen tissue, including blood vessels, smooth muscle and other stromal elements. They are characterized by a mixture of normal splenic tissue components arranged in an aberrant fashion, and have also been referred to as splenoma, splenadenoma, or nodular hyperplasia (12).

They are often asymptomatic and incidentally discovered during imaging studies. Although hamartomas are typically benign, they can occasionally present with symptoms due to their size or haemorrhagic complications, such as rupture, particularly in the context of splenic trauma or underlying medical conditions, as in the case presented here.

Seyama et al. (11) described a 53-year-old female with hepatitis C-related cirrhosis and SSR from a hamartoma, identified radiologically and confirmed histopathologically post-splenectomy. Barnes et al. (10) reported a 76-year-old male on warfarin presenting with SSR from a hamartoma, with CT showing active extravasation. Di Blasi et al. (13) and Yu et al. (12) characterised incidentally detected hamartomas, detailing radiological and histopathological features, though rupture was not observed in these cases.

### Frequency and prevalence

Splenic hamartomas are considered to be rare, accounting for less than 1% of all splenic lesions (2, 14). They usually are more common in adults and in individuals with a history of certain hematological disorders, such as lymphoproliferative diseases (15).

### Imaging characteristics

Ultrasound: typically well-defined, hypoechoic masses with variable internal echogenicity. Their heterogeneous appearance and possible isoechogenicity to splenic parenchyma can make diagnosis challenging.

CT: on contrast-enhanced CT, hamartomas often present as well-circumscribed lesions with heterogeneous enhancement due to vascular components. The enhancement pattern may overlap with that of other benign lesions such as haemangiomas or lymphangiomas, making clinical and laboratory correlation essential. They generally show inhomogeneous contrast enhancement during arterial and venous phases, with prevalent delayed enhancement in the equilibrium phase, a characteristic feature.

MRI: hamartomas typically show low-to-intermediate signal intensity on T1-weighted images and high signal intensity on T2-weighted images, reflecting their stromal and vascular content. Post-contrast sequences usually demonstrate heterogeneous enhancement.

Distinguishing hamartomas from other splenic lesions, such as metastases, lymphoma, or haemangiomas, can be challenging.

Histopathological confirmation is often required when the imaging findings are inconclusive (3, 12, 16).

## The role of radiology in diagnosis and management

CT imaging remains the gold standard for detecting splenic lesions and evaluating their extent. However, small or ambiguous lesions may require additional imaging and clinical correlation to ensure an accurate diagnosis. Ultrasound is widely accessible and cost-effective, but its diagnostic utility is limited by bowel gas artefacts and the isoechogenic appearance of some lesions relative to splenic parenchyma. CECT provides superior sensitivity for detecting splenic lesions and assessing associated complications, such as haemoperitoneum. Several case reports have demonstrated the efficacy of CT imaging in evaluating splenic size, hematoma formation, and intraperitoneal bleeding. For example, Nadaraja et al. (17) reported CT findings of an enlarged spleen with heterogeneous hypodensity and areas of enhancement corresponding to hematomas in a patient with pancreatitis. Lin JL et al. (18) described a spontaneous rupture of the spleen caused by an intra-splenic haemangioma, which was identified on CT as a hypervascular lesion associated with signs of hemoperitoneum. Management required a two-step approach: initial conservative treatment with embolisation of splenic branches, followed by splenectomy due to worsening haemoperitoneum on serial follow-up.

## Non-operative management (NOM): the preferred first-line approach

Historically, radiologists were primarily tasked with determining whether emergency conditions required surgical exploration. Nowadays, the concept of non-operative management (NOM) has become central. Many conditions that were once considered exclusively surgical are now graded to define the most appropriate treatment, and lower-grade injuries can be initially managed non-operatively. In this context, imaging plays an essential role that extends beyond grading; it is also crucial for reassessing the success or failure of NOM. Consequently, imaging has acquired an increasingly clinical and decision-support role in patient management.

NOM is the preferred approach in haemodynamically stable patients, aiming to reduce the morbidity associated with emergency surgery. Radiological monitoring is crucial for detecting early signs of clinical deterioration, such as increasing haemoperitoneum or contrast extravasation, which may necessitate intervention (19).

Advanced imaging, particularly CECT, is pivotal in assessing the severity of splenic injury, guiding clinical decision-making, and stratifying patients for conservative or surgical treatment. A key advantage of CECT lies in its ability to characterise active bleeding patterns—distinguishing minimal bleeding (spot sign), moderate extravasation (jet sign), and extensive haemorrhage (contrast pooling). The spot sign, indicative of limited haemorrhage, often suggests a favourable prognosis with a high likelihood of successful conservative management. Conversely, the jet sign and extensive pooling of contrast medium, indicating ongoing and significant haemorrhage, typically require a more aggressive approach, including angioembolisation or surgery (20, 21).

The radiologist plays an integral role within the multidisciplinary team managing splenic injuries, collaborating closely with clinicians and surgeons to tailor the management strategy to each patient. By providing detailed imaging assessments, radiologists help determine the urgency and type of intervention, thereby optimising outcomes (22, 23).

In both spontaneous and traumatic splenic rupture, an individualised, patient-specific approach is essential, ensuring timely and appropriate escalation of care when required. This personalised strategy, guided by imaging findings, allows more precise allocation of therapeutic resources, improving survival rates while minimising unnecessary surgical morbidity (24, 25). Marmery et al. (24) reported that in blunt splenic injury, NOM was pursued in 89% of patients (355/400). Of these, 341 (96%) were successfully treated without splenectomy, yielding an overall splenic salvage rate of 85% (341/400). For high-grade injuries (grades 3–5), NOM achieved a 95% success rate (136/143), corresponding to an overall splenic salvage rate of 76%. Osman et al. (26), reviewing 58 cases of SSR associated with malaria infection, reported that 19 patients were managed non-operatively with a 68% success rate (13/19). Including two additional cases from Khartoum (27), the overall success rate increased to 71% (15/21). Non-operative therapy failed in six patients (31%): four underwent splenectomy and survived, while two (11%) died without surgical intervention. Among those selected for operative management, the success rate was 93%.

In our case, although haemodynamic stability initially permitted NOM, current evidence indicates that anticoagulation is a significant predictor of NOM failure. Dougherty et al. (28) showed that trauma patients receiving anticoagulation had a markedly higher rate of splenectomy (23.3% vs. 11.6%) and an increased risk of NOM failure (20% vs. 0.7%) compared with non-anticoagulated patients. In light of these findings, our multidisciplinary team judged that elective surgical management would reduce the risk of delayed haemodynamic deterioration and emergency surgery. Moreover, the presence of an underlying lesion at the site of splenic rupture necessitated histopathological characterisation, which could not be achieved through conservative management. For these reasons, an elective splenectomy was undertaken as the most appropriate therapeutic strategy, balancing the risks of continued observation against the benefits of timely surgical intervention, in accordance of the best available evidence and tailored to the individual patient's clinical context.

## Management strategies and outcomes

**Figure 6:**
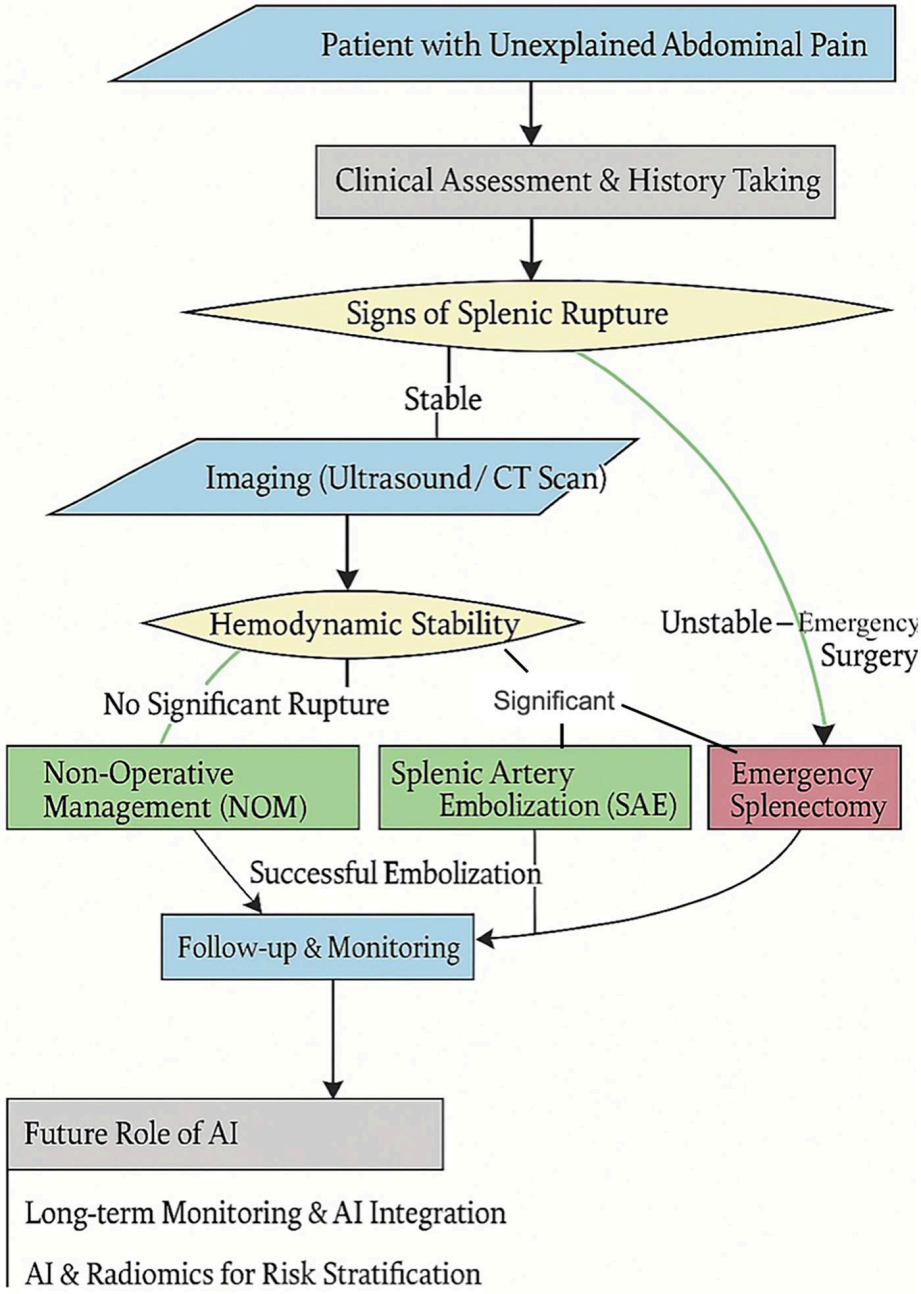
Clinical-radiological decision flowchart and operative and non-operative management in patients with SSR.

To control severe haemorrhagic complications urgent splenectomy remains the prevailing therapeutic approach for managing SSR, as demonstrated in cases reported by Tunçyürek et al. (5), Roll et al. (7), Looseley et al. (8), Seyama et al. (11), and Barnes et al. (10). Chun et al. (26) showcased the efficacy of splenic artery embolisation as a minimally invasive alternative, particularly suitable for patients maintaining haemodynamic stability. The importance of early CT imaging, as emphasised by Tunçyürek et al. (5) and Ballardini et al. (6), cannot be overstated, as it markedly improves patient prognosis by enabling swift and targeted interventions. In Figure 6 a flowchart of our production for the diagnosis and manager of the SSR.

## The emerging role of artificial intelligence and radiomics in splenic imaging

AI and radiomics are emerging technologies with substantial potential in the early detection and management of splenic pathologies. Radiomics involves extraction of high-dimensional quantitative features from medical images, providing a deeper understanding of tissue characteristics such as texture, shape, intensity distribution, and wavelet-based parameters. These features may reveal subtle differences in tissue composition that are not visually discernible to radiologists (3, 4).

In splenic pathology, radiomics can be used to:

Differentiate between benign and malignant splenic lesions by characterising heterogeneity and vascular patterns.
Predict the risk of splenic rupture by evaluating structural integrity and parenchymal fragility.
Enhance early detection of haematologic disorders, where changes in splenic texture and density may serve as imaging biomarkers (e.g., in lymphoma or leukaemia).
Improve risk stratification and treatment planning when integrated with clinical and laboratory data.

Recent research suggests that machine learning models trained on radiomic data can achieve high accuracy in distinguishing normal from pathological spleens, with reported accuracies exceeding 85%, outperforming conventional imaging interpretation in some settings (29).

A recent study developed a hybrid deep learning framework for spleen segmentation and trauma detection in ultrasound images, addressing challenges such as low contrast, noise, and anatomical variability. Using transfer learning from porcine to human data, the AI model detected splenic trauma on standard ultrasound, achieving up to 90% AUC and outperforming two experienced radiologists in both sensitivity and specificity. This approach offers a fast, non-invasive aid for trauma assessment, particularly valuable where CT or contrast-enhanced ultrasound (CEUS) are not available, although further multicentre validation is required (30).

## Conclusion

Spontaneous splenic rupture is a rare but critical condition that must be considered in patients presenting with unexplained abdominal pain and underlying comorbidities. Timely and accurate diagnosis requires a multidisciplinary approach, in which radiologists and imaging play an increasingly important role in decision-making and treatment selection. Emerging technologies such as AI and radiomics are poised to further enhance diagnostic accuracy and improve patient outcomes. Future research should focus on refining AI-driven approaches for splenic imaging, optimising risk stratification, and enabling more personalised patient care.
